# Predictors of seizure outcomes in patients with diffuse low‐grade glioma‐related epilepsy after complete glioma removal

**DOI:** 10.1111/cns.14061

**Published:** 2022-12-13

**Authors:** Xinghui He, Kai Zhang, Dingyang Liu, Zhuanyi Yang, Xuejun Li, Zhiquan Yang

**Affiliations:** ^1^ Department of Neurosurgery, Xiangya Hospital Central South University Changsha China

**Keywords:** diffuse low‐grade glioma, diffuse low‐grade glioma‐related epilepsy, epilepsy, seizure outcomes

## Abstract

**Aims:**

We aimed to identify predictors of postoperative seizures in patients with diffuse low‐grade glioma (DLGG)‐related epilepsy after complete tumor resection in this study.

**Methods:**

We retrospectively collected data from individuals with DLGG‐related epilepsy whose tumors were completely resected at Xiangya Hospital, Central South University between January 2014 and January 2020. The predictors of seizure outcomes were assessed by employing univariate analysis and a multivariate logistic regression model in a backward binary logistic regression model.

**Results:**

Among the 118 cases that met the inclusion criteria, 83.05% were seizure‐free following an average follow‐up of 4.27 ± 1.65 years, all of whom were classified as International League Against Epilepsy class I outcome. Univariate and multivariate analyses indicated that seizure duration of >6 years (odds ratio [OR], 6.62; 95% confidence interval [CI], 1.76–24.98; *p* = 0.005) and first clinical symptoms other than seizures (OR, 4.51; 95% CI, 1.43–14.23; *p* = 1.010) were both independent predictors of unfavorable seizure outcomes.

**Conclusion:**

Our results imply that satisfactory seizure outcomes can be achieved in most patients with DLGG‐related epilepsy after complete tumor resection. Patients with seizure duration of >6 years or first clinical symptoms other than seizures were more likely to experience postoperative seizure recurrence.

## Graphical abstract

Our study revealed that seizure duration greater than 6 years or first clinical symptoms other than seizures were independent predictors of unfavorable seizure outcomes in patients with diffuse low‐grade glioma‐related epilepsy after complete tumor removal.

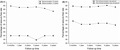

## 1 INTRODUCTION

Up to 80% of patients with diffuse low‐grade gliomas (DLGG, WHO grade II glioma) are accompanied by epileptic seizures.^1^, ^2^ Epilepsy related to DLGG tends to be focal, with or without focal‐to‐bilateral tonic–clonic seizures, and is always resistant to antiepileptic medication.^3^, ^4^ Persistent uncontrolled seizures have a negative influence on life quality and oncological outcomes, consequently increasing the financial and psychosocial burden on patients and their families.^5^, ^6^ Thus, both oncological and seizure control are important goals for DLGG treatment.

Resection surgery is presently considered the most effective treatment for seizure control in patients with DLGG‐related epilepsy.^1^, ^7^ The extent of tumor excision is one of the most important predictors of seizure control after operation and is partly attributed to the neurotransmitter released by the residual tumor.^8^, ^9^, ^10^ Accordingly, maximal safe resection of tumors is recommended as the standard of care for DLGG‐related epilepsy.^11^ However, postoperative seizures persist in nearly 20% of patients even after complete tumor resection,^2^, ^9^, ^12^ implying that a detailed preoperative evaluation of epileptogenic focus in a specialized epilepsy center may benefit these patients. Identifying the predictors of seizure outcomes after complete tumor excision may help improve patient selection and management during the perioperative period. Although numerous studies have examined predictors of seizure outcomes in patients with DLGG‐related epilepsy after surgery, the predictors of seizure outcomes after complete tumor resection remain elusive; previous studies included patients with different extents of tumor resection.^2^, ^8^, ^9^, ^12^, ^13^

Here, we report 118 cases of DLGG‐related epilepsy whose tumors were completely removed. The objective of this study was to analyze the predictors of seizure outcome after complete resection of DLGG, which may useful in selection of patients with DLGG‐related epilepsy who would be candidates for detailed preoperative evaluation, or postoperative medical management to monitor for and address seizure activity.

## 2 METHODS

Ethics authorization was acquired by the Xiangya Hospital Ethics Committee of Central South University, and written informed consent was collected from all participants or their legal guardians.

### 2.1 Patient selection

We retrospectively collected clinical data on individuals with DLGG‐related epilepsy who experienced resection operation at the Department of Neurosurgery, Xiangya Hospital, Central South University between January 2014 and January 2020. The inclusion criteria were as follows: (1) patients with supratentorial DLGG (WHO grade II astrocytoma or oligodendrocytoma^14^) identified by postoperative pathological diagnosis, (2) patients with preoperative focal epilepsy associated with DLGG, (3) patients who had undergone surgical treatment for the first time, (4) patients whose postoperative magnetic resonance imaging (MRI) revealed complete DLGG removal; and (5) Postoperative patients who have been observed for more than 2 years.

**FIGURE 1:**
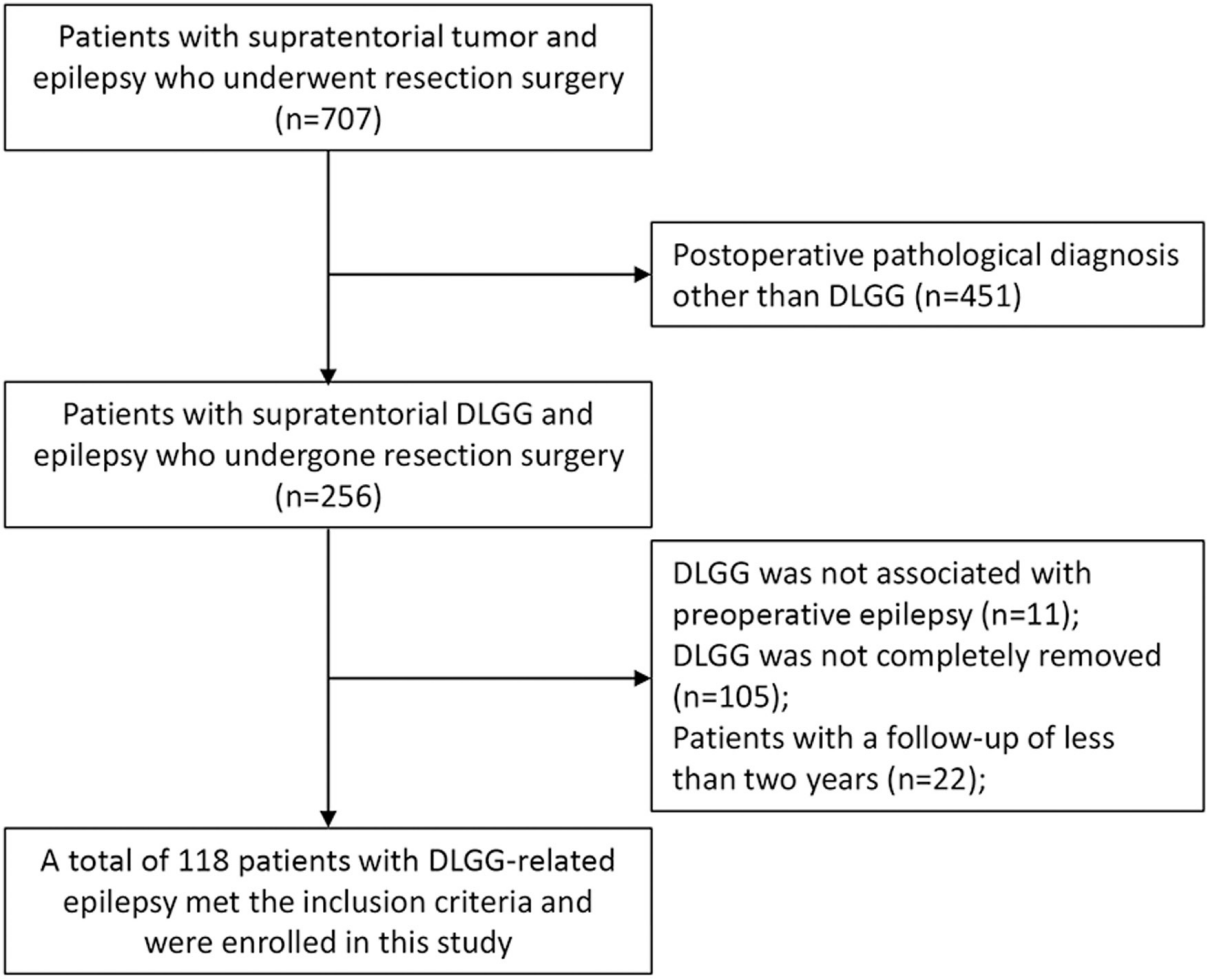
Flow diagram of patients. DLGG, diffuse low‐grade glioma

The following were the criteria for exclusion: (1) patients who had pathological diagnosis other than DLGG; (2) patients who did not undergo resection surgery, or in whom the tumor was not completely removed; (3) patients without preoperative epilepsy, or in whom the DLGG was not associated with preoperative epilepsy according to preoperative evaluations; and (4) individuals with fewer than 2 years of follow‐up. Figure 1 illustrates the flow chart of patients.

### 2.2 Data collection and evaluation

Clinical data, including age at operation, age at seizure onset, epileptic semiology, seizure duration, seizure rate, histopathologic diagnosis, lesion location, and treatment information, were obtained from department of clinical records. The size of the DLGG was evaluated based on preoperative MRI. Postoperative MRI was performed either 72 h or 3 months following the surgery in all participants. Independent neurosurgeons evaluated the extent of resection depending on the T2 weight fluid‐attenuated inversion recovery sequency MRI scan and detected it as: (tumor volume before surgery‐tumor volume after surgery)/preoperative tumor volume.

### 2.3 Follow‐up and assessment of surgical outcomes

There was at least a 2‐year follow‐up for all individuals. Using the International League Against Epilepsy (ILAE) classification,^15^ neurosurgeons assessed seizure outcomes either in the outpatient service or by telephone interviews. Classes 1 and 2 of the ILAE were assigned to good seizure outcomes, whereas classes 3 to 6 were assigned to poor results.

### 2.4 Statistical analysis

Continuous variables are expressed as mean ± standard deviation (SD), while percentages are employed for categorical variables. For seizure outcome analyses, patients were classified as having either favorable or unfavorable seizure outcomes along with the last follow‐up.

Continuous variables were first cut off according to Youden's index in receiver operating characteristic curve analysis. For univariate analysis, Pearson's chi‐squared test or Fisher's exact assessment was used. After the univariate analysis, variables with a *p*‐value of <0.2 were recruited into a backward binary logistic regression model to analyze the independent predictors. SPSS version 22 (IBM, Armonk, NY, USA) was employed for all statistical analyses. Statistical significance was recognized at a *p*‐value of <0.05.

## 3 RESULTS

### 3.1 Patient features

One hundred and eighteen DLGG‐related epilepsy individuals met the inclusion criteria and were enrolled in this investigation. Of these, 63 (53.39%) were male and 55 (46.61%) were female. The mean age at surgery was 33.14 ± 13.79 years, mean age at seizure onset was 31.30 ± 14.71 years, and mean seizure duration (time span between first seizures and surgery) was 1.9 ± 4.23 years. Other characteristics are summarized in Table 1.

**TABLE 1:** Clinical characteristics of patients with DLGG‐related epilepsy in the present study (*n* = 118)

Clinical characteristics	Value
Sex, *n* (%)	
Male	63 (53.39)
Female	55 (46.61)
Age at surgery, mean ± SD years	33.14 ± 13.79
Age at seizure onset, mean ± SD years	31.30 ± 14.71
Seizure duration, mean ± SD years	1.89 ± 4.23
Monthly seizure frequency, mean ± SD times	5.71 ± 14.24
First clinical symptom, *n* (%)	
Seizure	95 (80.51)
headache	15 (12.71)
dizziness	3 (2.54)
vomiting	1 (0.85)
Headache and vomiting	1 (0.85)
Headache and dizziness	1 (0.85)
paresthesia	1 (0.85)
memory deterioration	1 (0.85)
Seizure Types, *n* (%)	
Focal only	32 (27.12)
Focal‐to‐bilateral tonic–chronic seizures	86 (72.88)
Auras, *n* (%)	
Yes	16 (13.56)
No	102 (86.44)
Preoperative SE, *n* (%)	
Yes	5 (4.24)
No	113 (95.76)
Side of tumors, *n* (%)	
Right	57 (48.31)
Left	61 (51.69)
Site of tumors, *n* (%)	
Frontal lobe	53 (44.92)
Temporal lobe	33 (27.97)
Parietal lobe	8 (6.78)
Insular lobe	1 (0.85)
Multilobar	23 (19.49)
Size of tumor, mean ± SD cm	4.56 ± 1.56
Pathology, *n* (%)	
Astrocytoma	81 (68.64)
Oligodendroglioma	37 (31.36)

Abbreviations: SD, standard deviation; SE, status epilepticus.

### 3.2 Preoperative evaluation, treatment, and histopathology

**FIGURE 2:**
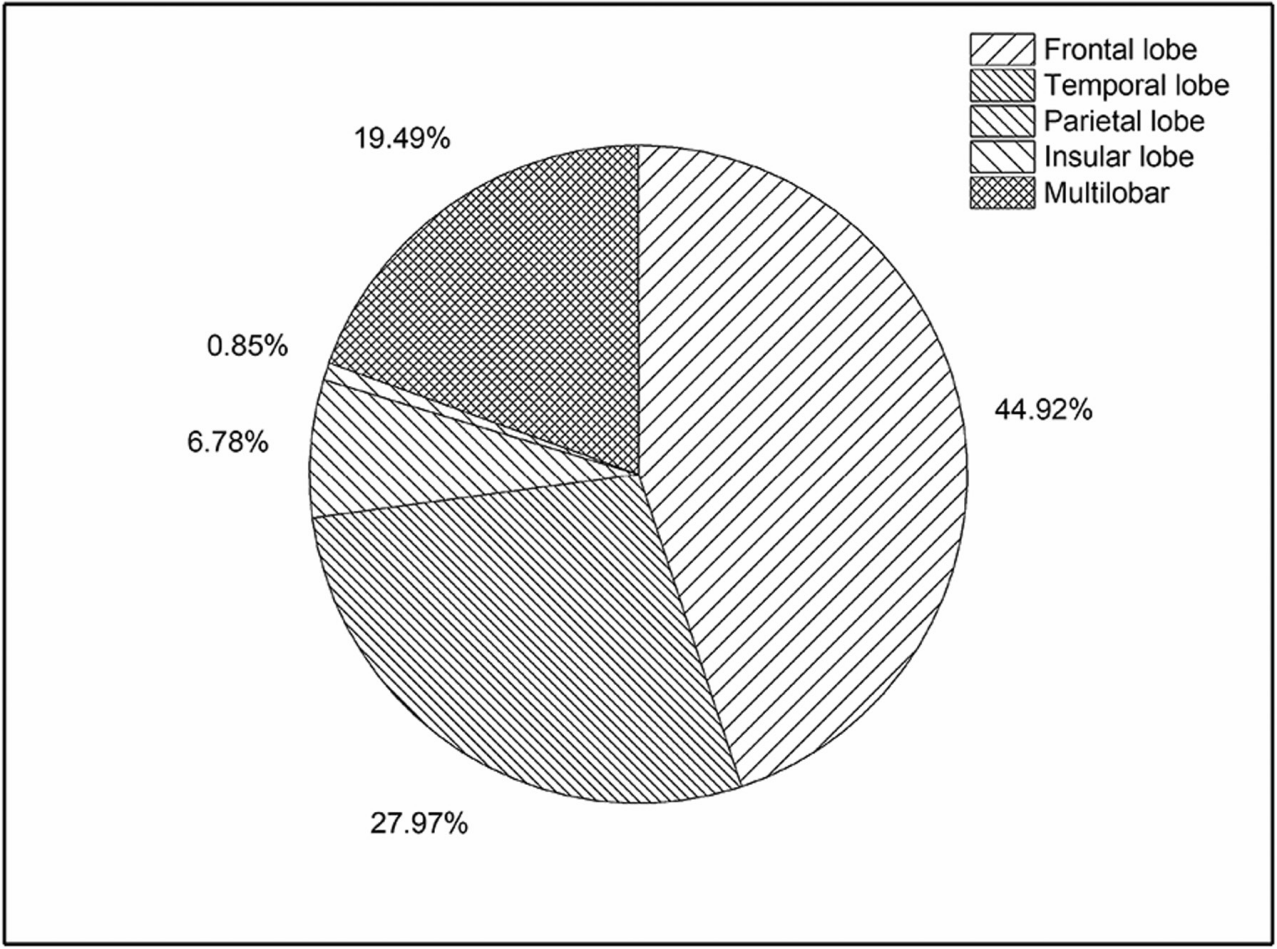
Patient distribution according to tumor locations

Brain MRI with T1‐weighted sequences, T2‐weighted sequences, fluid‐attenuated inversion recovery sequences, and contrast enhancement MRI on a 1.5 or 3.0‐T scanner were carried out in all participants preoperatively for tumor assessment. Figure 2 shows patient distribution according to tumor location. To identify the correlation between abnormal epileptiform discharges and tumors, a two‐hour scalp electroencephalogram was performed in all patients, and a long‐term video electroencephalogram was additionally performed in 44 (37.29%) patients.

Resection surgery was performed according to the findings of preoperative evaluations in all patients; 22 patients (18.64%) underwent intraoperative electrocorticography (ECoG). Histopathology confirmed the tumor as WHO grade II astrocytoma in 81 (68.64%) patients and as WHO grade II oligodendroglioma in 37 (31.36%) patients. Molecular analyses of *IDH1* (isocitrate dehydrogenase 1) were performed in 114 (96.61%) patients, and *IDH1* mutations were observed in 89 (75.42%) patients.

All patients routinely received antiepileptic drug (AED) therapy for at least 6 months after surgery. For postoperative oncology treatment, 50 (42.37%) patients received postoperative radiotherapy and 49 (41.53%) underwent postoperative chemotherapy.

### 3.3 Surgical outcomes and complications

With a mean follow‐up of 4.27 ± 1.65 years, 98 (83.05%) patients were seizure‐free. The results of seizures are shown in Table 2 according to the duration of follow‐up. At the last follow‐up, AEDs were discontinued in 53 patients who were seizure‐free (44.92%). Tumor recurrence was observed in 6 (5.08%) patients during follow‐up, among whom seizure recurrence occurred in 3 (2.54%) patients. Patients with seizure recurrence showed a significantly elevated risk of tumor recurrence (*X* ^2^ = 73.51; *p* < 0.001).

**TABLE 2:** Seizure outcomes according to follow‐up period

ILAE classification	1	2	3	4	5	6
3 months, *n* = 118	101 (85.59)	0 (0.00)	1 (0.85)	9 (7.63)	6 (5.08)	1 (0.85)
1 year, *n* = 118	99 (83.90)	0 (0.00)	2 (1.69)	10 (8.47)	6 (5.08)	1 (0.85)
2 years, *n* = 118	99 (83.90)	0 (0.00)	3 (2.54)	9 (7.63)	6 (5.08)	1 (0.85)
3 years, *n* = 104	88 (84.62)	0 (0.00)	2 (1.92)	8 (7.41)	5 (4.81)	1 (0.96)
4 years, *n* = 70	59 (84.29)	0 (0.00)	1 (1.43)	4 (5.71)	5 (7.14)	1 (1.43)
5 years, *n* = 48	39 (81.25)	0 (0.00)	1 (2.08)	3 (6.25)	4 (8.33)	1 (2.08)
Last follow‐up, *n* = 118	98 (83.05)	0 (0.00)	3 (2.54)	10 (8.47)	6 (5.08)	1 (0.85)

*Note*: Data were shown as *n* (%) of patients.

There were 12 (10.17%) patients with surgical complications: hemiparesis occurred in 10 (8.47%) patients, Broca's aphasia occurred in 1 (0.85%), and both hemiparesis and Broca's aphasia in 1 (0.85%). Five (4.24%) patients recovered completely during follow‐up, and permanent mild hemiparesis was observed in the remaining seven (5.93%) patients. No perioperative mortality was observed in this study.

### 3.4 Predictors of seizure outcomes

**FIGURE 3:**
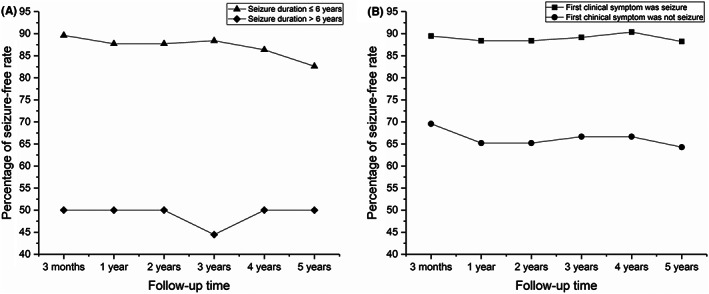
Seizure‐free rate at different follow‐up time points according to independent predictors, including seizure duration (A) and the first clinical symptom (B).

Seizure activity reported at the final follow‐up was analyzed to assess predictors of seizure outcomes. In univariate analysis, seizure duration and first clinical symptoms were substantially related to postoperative seizure outcomes, and factors including performance of intraoperative ECoG, *IDH1* mutation, and seizure types illustrated a *p*‐value of <0.2 (Table 3). These five factors were incorporated in the backward binary logistic regression model. Multivariate analysis showed that seizure duration greater than 6 years (odds ratio [OR], 6.62; 95% confidence interval [CI], 1.76–24.98; *p* = 0.005) and first clinical symptoms other than seizures (OR, 4.51; 95% CI, 1.43–14.23; *p* = 1.010) were independent predictors of unfavorable seizure outcomes (Table 4). Figure 3 illustrates seizure outcomes according to independent predictors at various follow‐up points.

**TABLE 3:** Clinical characteristics and their relationship with seizure outcomes in 118 patients with DLGG‐related epilepsy after complete tumoral removal

Variables	Favorable seizure outcomes	Unfavorable seizure outcomes	*p*‐value
Sex			
Male	50 (42.37)	13 (11.02)	0.253
Female	48 (40.68)	7 (5.93)	
Age at surgery			
≤30 years	43 (36.44)	6 (5.08)	0.251
>30 years	55 (46.61)	14 (11.86)	
Age at seizure onset			
≤30 years	49 (41.53)	7 (5.93)	0.221
>30 years	49 (41.53)	13 (11.02)	
Seizure duration			
≤6 years	92 (77.97)	14 (11.86)	0.005^b^*
>6 years	6 (5.08)	6 (5.08)	
Seizure frequency			
Monthly	73 (61.86)	16 (13.56)	0.813^b^
Sparse	25 (21.19)	4 (3.39)	
First clinical symptom			
Seizures	83 (70.34)	12 (10.17)	0.026^b^*
Others	15 (12.71)	8 (6.78)	
Seizure Types			
Focal only	29 (24.58)	3 (2.54)	0.181
Focal‐to‐bilateral tonic–chronic seizures	69 (58.47)	17 (14.41)	
Auras			
Yes	14 (11.86)	2 (1.69)	0.879^b^
No	84 (71.19)	18 (15.25)	
Preoperative SE			
Yes	5 (4.24)	0 (0.00)	0.587^c^
No	93 (78.81)	20 (16.95)	
Side of tumors			
Left	52 (44.07)	9 (7.63)	0.511
Right	46 (38.98)	11 (9.32)	
Site of tumors			
Frontal lobe	43 (36.44)	10 (8.47)	0.242
Temporal lobe	28 (23.73)	5 (4.24)	
Parietal lobe	7 (5.93)	1 (0.85)	
Insular lobe	0 (0.00)	1 (0.85)	
Multilobar	20 (16.95)	3 (25.42)	
Size of tumor			
<4 cm	33 (27.97)	5 (4.24)	0.449
≥4 cm	65 (55.08)	15 (12.71)	
Performance of preoperative VEEG			
Yes	35 (29.66)	9 (7.63)	0.434
No	63 (53.39)	11 (9.32)	
Performance of intraoperative ECoG			
Yes	21 (17.80)	1 (0.85)	0.160^b^
No	77 (65.25)	19 (16.10)	
Surgical Complications			
Yes	12 (10.17)	0 (0.00)	0.213^b^
No	86 (72.88)	20 (16.95)	
Pathology			
Astrocytoma	65 (55.08)	16 (13.56)	0.23
Oligodendroglioma	33 (27.97)	4 (3.39)	
IDH mutation			
Yes	73 (61.86)	16 (13.56)	0.101
No	23 (19.49)	2 (1.69)	
NOS	2 (1.69)	2 (1.69)	
Acute postoperative seizures^a^			
Yes	12 (10.17)	2 (1.69)	>0.999^b^
No	86 (72.88)	18 (15.25)	
Postoperative chemotherapy			
Yes	40 (33.90)	9 (7.63)	0.729
No	58 (49.15)	11 (9.32)	
Postoperative radiotherapy			
Yes	42 (35.59)	8 (6.78)	0.814
No	56 (47.46)	12 (10.17)	

Abbreviations: ECoG, electrocorticography; SE, status epilepticus; VEEG, video electroencephalogram.

*p < 0.05.

^a^ Seizures occurred during the first week after surgery.

^b^ For comparisons of binary variables, chi‐squared test with continuity correction was used.

^c^ For comparisons of binary variables, Fisher's exact test was used.

**TABLE 4:** Predictors of postoperative seizure outcome in patients with DLGG‐related epilepsy after complete tumoral removal on multivariate analysis

Variables	OR	95% CI	*p*‐value
Seizure duration (>6 years)	6.62	1.76–24.98	0.005*
First clinical symptom (other than seizures)	4.51	1.43–14.23	0.010*
Performance of intraoperative ECoG (No)	6.49	0.69–61.33	0.102
IDH1 mutation			
Yes	0.26	0.020–2.79	0.263
No	0.09	0.01–1.40	0.085
Seizure Types (Focal only)	0.45	0.11–1.90	0.278

Abbreviation: EcoG, electrocorticography.

*p < 0.05.

## 4 DISCUSSION

DLGG is among the most epilepsy‐causing brain injuries; however, patients have long life span.^16^, ^17^, ^18^ Identifying the predictors of postoperative seizure outcomes is important for clinical management. The extent of tumor resection is one of the most significant predictors of seizure control following surgery in individuals with DLGG‐related epilepsy according to some previous studies.^8^, ^9^, ^10^ However, persistent seizures exist in some patients, even after complete tumor resection,^2^, ^9^, ^12^ and predictors of seizure outcomes after complete DLGG resection are poorly understood. In the current investigation, a large case series of 118 participants who underwent complete resection of their tumors with DLGG‐related epilepsy was analyzed for the predictors of seizure outcomes.

Seizure‐free status was obtained in 83.05% of cases at the last follow–up, which is slightly higher than the findings of previous studies indicating that 64.5%–82.0% of patients achieved seizure freedom after tumor resection.^10^, ^12^, ^19^, ^20^ This may be because only patients with complete tumor resection were included in our research. Complications following the operation happened in 10.17% of cases, and permanent neurological impairments occurred in 5.93% of patients, similar to previous studies on surgical treatment for DLGG.^2^, ^8^ Notably, in this case series, patients who experienced surgical complications were seizure‐free at the final follow‐up. This implies a balance between seizure control and surgical risks in some patients. Seizure recurrence has been informed to be related to tumor recurrence.^21^, ^22^ In accordance with the aforementioned studies, we observed tumor recurrence in 5.08% of individuals in this study, and during follow‐up, half of these individuals suffered a seizure recurrence.

Identifying the predictors of seizure outcomes in DLGG‐related epilepsy individuals after complete tumor resection is essential, as seizures significantly reduce the quality of life. Furthermore, knowing the predictors may help during candidate selection for detailed preoperative evaluation of the epileptogenic zone.^2^ In our study, seizure duration of ≥6 years and the first clinical symptoms other than seizures were independent predictors of seizure recurrence in DLGG‐related epilepsy individuals after complete tumor resection.

Similar to our findings, the relation between extended seizure duration and unfavorable seizure outcomes has been noted in previous studies.^8^, ^20^, ^23^ Long‐term uncontrolled seizures may cause a more complicated epileptogenic network or formation of a second epileptogenic focus.^24^, ^25^, ^26^ In patients with DLGG‐related epilepsy, microenvironmental and functional changes in the peritumoral tissue caused by tumor progression may also contribute.^1^, ^27^ Thus, early surgery may benefit both oncological and seizure control. In this study, we identified the appropriate time for surgical intervention. Our data imply that a detailed assessment of epileptogenic focus may provide additional benefits for patients with seizure durations of >6 years.

Interestingly, we also found that the first clinical symptom other than seizures was another independent predictor of unfavorable seizure outcomes, which was not reported in previous studies. In DLGG‐related epilepsy, the origin and mechanisms are multifactorial and intermixed,^1^, ^28^ suggesting that the main epileptogenic factors vary among individuals, even in patients with similar pathological diagnoses. In patients whose first clinical symptom is seizures, the neurotransmitters or modulators released by the tumor may play the main role in epileptogenesis,^1^, ^29^ while structural reorganization or functional changes within the peritumoral tissue may be mainly responsible for seizures in patients with first clinical symptoms other than seizures.^27^, ^30^, ^31^

## 5 LIMITATIONS

This study has some limitations. First, inherent biases cannot be disregarded due to the single‐center retrospective design of this study. Second, the preoperative evaluations were not all standard (e.g., video electroencephalogram monitoring was not performed in all patients), which may reflect the reality of daily clinical practice. Third, although it was not a goal of the present study, changes in patients' quality of life were not assessed, which may also be a goal of surgical treatment. Even though these restrictions, our data offer valuable information about the predictors of postoperative seizure outcomes in patients with DLGG‐related epilepsy after complete tumor resection, which may be useful in clinical practice.

## 6 CONCLUSIONS

Our data reveal that satisfactory seizure outcomes can be achieved in most patients with DLGG‐related epilepsy after complete tumor resection. Patients with a seizure duration of >6 years or with first clinical symptoms other than seizures were more likely to experience postoperative seizure recurrence.

## CONFLICT OF INTEREST

The authors report having no conflicts of interest.

## Data Availability

The datasets created and assessed during the present investigation are accessible upon reasonable request from the corresponding author.
